# Notch4 is required for tumor onset and perfusion

**DOI:** 10.1186/2045-824X-5-7

**Published:** 2013-04-20

**Authors:** Maria José Costa, Xiaoqing Wu, Henar Cuervo, Ruchika Srinivasan, Seth K Bechis, Ellen Cheang, Olivera Marjanovic, Thomas Gridley, Christin A Cvetic, Rong A Wang

**Affiliations:** 1 Laboratory for Accelerated Vascular Research, Division of Vascular Surgery, Department of Surgery, University of California, San Francisco, CA 94143, USA; 2 Center for Molecular Medicine, Maine Medical Center Research Institute, 81 Research Drive, Scarborough, ME 04074, USA; 3 Present address: Department of Pediatrics and Program in Regenerative Medicine, Stanford University, Stanford, CA 94305, USA; 4 Present address: Tech Data Services, LLC, King of Prussia, PA19406, USA; 5 Present address: Novartis Healthcare Pvt. Ltd., Hyderabad, India; 6 Present address: Department of Urology, Massachusetts General Hospital, Boston, MA 02114, USA; 7 Present address: Department of Radiology, University of California Davis Medical Center, Sacramento, CA 95817, USA; 8 Present address: School of Public; Division of Infectious Diseases and Vaccinology, University of California Davis Medical Center, Sacramento, CA 95817, USA

**Keywords:** Notch, Angiogenesis, Tumor, Blood vessel, Perfusion

## Abstract

**Background:**

Notch4 is a member of the Notch family of receptors that is primarily expressed in the vascular endothelial cells. Genetic deletion of *Notch4* does not result in an overt phenotype in mice, thus the function of Notch4 remains poorly understood.

**Methods:**

We examined the requirement for Notch4 in the development of breast cancer vasculature. Orthotopic transplantation of mouse mammary tumor cells wild type for *Notch4* into *Notch4* deficient hosts enabled us to delineate the contribution of host Notch4 independent of its function in the tumor cell compartment.

**Results:**

Here, we show that Notch4 expression is required for tumor onset and early tumor perfusion in a mouse model of breast cancer. We found that Notch4 expression is upregulated in mouse and human mammary tumor vasculature. Moreover, host *Notch4* deficiency delayed the onset of *MMTV-PyMT* tumors, wild type for *Notch4*, after transplantation. Vessel perfusion was decreased in tumors established in *Notch4-*deficient hosts. Unlike in inhibition of Notch1 or Dll4, vessel density and branching in tumors developed in *Notch4*-deficient mice were unchanged. However, final tumor size was similar between tumors grown in wild type and Notch4 null hosts.

**Conclusion:**

Our results suggest a novel role for Notch4 in the establishment of tumor colonies and vessel perfusion of transplanted mammary tumors.

## Background

Notch signaling underlies an evolutionarily conserved mechanism that regulates a wide range of cellular processes via direct cell-cell communication. In mammals, there are five Notch ligands (Jagged1, Jagged2, Delta-like 1/Dll1, Dll3, and Dll4) and four Notch receptors (Notch1-4). The interaction between the Notch ligand and its receptor triggers two proteolytic cleavages that release the Notch intracellular domain into the nucleus. There, the Notch intracellular domain forms complexes with members of the CSL transcription factor family (CBF1/RBP-Jκ/Suppressor of Hairless/LAG-1), leading to the expression of downstream transcription factors (reviewed in [1]).

Among the four Notch receptors, Notch1 and Notch4 are expressed in the vascular endothelium, with Notch4 expression being more restricted to endothelial cells (ECs) [2]. Mice homozygous for a null allele of *Notch1* develop vascular abnormalities and die *in utero* shortly after primitive vascular plexus formation [3,4]. Homozygous *Notch4* null mice develop normally and are viable and fertile. However, the combination of homozygous loss of *Notch1* and *Notch4* results in a more pronounced vascular phenotype than the *Notch1* null homozygous alone [3,4]. Even though this finding suggests that lack of Notch4 can exacerbate the effect of Notch1 deficiency, the unique role for Notch4 in vascular development is unknown. In adult mice, genetic deletion of *Notch4* does not lead to any detectable abnormalities, other than a slightly elevated systolic blood pressure after experimental induction of hindlimb ischemia [3,5].

Angiogenesis, the formation of new vessels from pre-existing ones, plays a key role in cancer and other pathological conditions. Solid tumors depend on the development of blood vessels to provide oxygen and nutrients to support their growth [6]. Interfering with Notch1 signaling dramatically affects angiogenesis in the developing and pathological vasculatures (reviewed in [7,8]). In solid tumors, blocking Dll4 leads to excessive sprouting and “non-productive” angiogenesis, which results in decreased vessel perfusion and inhibition of tumor growth [9,10]. Additionally, selective blocking of Notch1 with antibodies inhibits tumor growth by a mechanism consistent with that of Dll4 inhibition, namely by promoting abnormal growth of vessel sprouts that do not perfuse tissues [11]. Thus while the importance of the Dll4-Notch1 signaling axis in tumor angiogenesis is well documented, whether Notch4, primarily expressed in the endothelium, is required for angiogenesis, vascular perfusion, and tumor progression is unknown.

To unravel the role of Notch4 in tumor angiogenesis, we chose breast cancer as a solid tumor model. Since Notch4 is predominantly expressed in the vascular endothelium in mice [2], we therefore used orthotopic transplantation of mouse mammary carcinoma cells wild type for *Notch4* into syngeneic *Notch4-*deficient hosts to test the importance of Notch4 in tumor angiogenesis.

## Methods

### Animals and human samples

C57BL/6J wild type mice were purchased from Jackson Laboratory*. MMTV-PyMT* transgenic mice in C57 background were provided by Dr. Zena Werb’s lab at the University of California, San Francisco (UCSF). All strains were bred and maintained in a C57BL/6J background. We crossed wild type mice to *Notch4*^−/−^ mice to obtain *Notch4*^+/−^ mice, which were then intercrossed to generate *Notch4*^−/−^ and *Notch4*^+/+^ controls. Mice were kept in a pathogen-barrier facility from the time of breeding to euthanasia.

Human breast cancer samples (paraffin-embedded tissue blocks) were obtained from the UCSF Breast Cancer SPORE tissue core, which collected samples in compliance with a protocol approved by the UCSF Committee on Human Research. Patient consent was obtained using the standard surgical consent form, in combination with the brochure, Donating Tissue for Medical Research. The research involved collection of left-over specimens and, as such, involved no more than minimal risk to the subjects. Our use of these tissues was also approved by the UCSF Committee on Human Research.

### Immunostaining

Paraffin tissue sections of human samples were processed using standard methods. Mouse tissue samples were fixed in 4% formaldehyde in phosphate-buffered saline (PBS), embedded in OCT (Tissue Tek), frozen, and cryosectioned. Immunostaining was performed using rabbit anti-Notch4 (Upstate, diluted 1/1000) and rat anti-mouse CD31 (MEC 13.3, Pharmingen, diluted 1/500) as previously described [12,13]. Secondary antibodies were Cy3-conjugated donkey anti-rabbit (diluted 1/1000) and alexa-488 donkey anti-rat (diluted 1/1000), both from Jackson ImmunoResearch. In experiments with mouse tissue tumors, sections were collected from three different regions of each tumor and quantification was performed from three different fields per section. When tumors were not detected by naked eye, the whole mammary fat pad was preserved for sectioning and microscopical analysis.

### Animal procedures

Animal experiments were performed in compliance with the guidelines of UCSF Institutional Animal Care and Use Committee (IACUC), who approved this study under protocol #AN085404 and AN075264. We routinely monitored our mice targeted for tumorigenesis before and during tumor formation. Mice that displayed any of the following signs were euthanized: loss of 15% of their initial body weight, tumor size of 2 cm in the largest dimension, or decline in activity.

### Isolation of primary tumor cells and orthotopic transplantation

Primary tumor epithelial cells were isolated from mammary carcinomas that developed in *MMTV-PyMT* mice as previously described [14]. Briefly, tumors were dissected from 4–5 month old females, minced with razor blades and collagenase-digested (Sigma Blend L 0.5 mg/ml in RPMI 1640, supplemented with 10 mM Hepes and 5% fetal bovine serum) for 1 hour at 37°C with continuous agitation. The resultant mammary epithelial “organoids” were washed in Hank’s medium with calcium and magnesium (with 5% fetal bovine serum), separated from single cells and blood through differential centrifugation, trypsinized for 20 minutes and treated with DNaseI to obtain single cell suspensions. These were immediately frozen at 5 × 10^6^ cells/vial, and preserved in liquid nitrogen for later use. Each cell aliquot was then thawed and used for transplants in paired sets of *Notch4*^−/−^ and control animals. Cells were washed and resuspended in ice-cold PBS. Mice were anesthetized using isoflurane and a ventral incision was made to expose the fourth inguinal mammary glands. Approximately 10^6^ cells (in a 10 μl volume) were injected into syngeneic C57BL/6J wild type or *Notch4*^−/−^ female mice at 3 weeks of age. Tumor development was monitored every other day by palpation.

### Vessel perfusion studies

Mice were anesthetized with isoflurane and injected in the tail vein with a combination of 60 μg of biotinylated *Lycopersicon esculentum* lectin (Vector Laboratories, Burlingame, CA) and 60 μg of Cy3-streptavidin (Jackson ImmunoResearch). Mice were further anesthetized with ketamine/xylazine. After 5 minutes, the chest was opened and the vasculature was perfused with PBS through the left ventricle for 5 minutes, followed by 4% formaldehyde in PBS for 3 minutes at a pressure of 100 mm Hg.

### Real-time PCR

Total RNA was isolated from normal mammary inguinal fat pads and from transplanted tumors using Trizol (Invitrogen), following the manufacturer’s instructions. RNA was further purified from contaminant genomic DNA with DNase I treatment and RNeasy columns (Qiagen), following the manufacturer’s instructions. RNA was retro-transcribed using SuperscriptIII (Invitrogen) and SYBR Green-based real-time-PCR was used to analyze gene expression. Normalized fold change in gene expression in tumor relative to normal gland was calculated according to Pffafl [15], using *Tie2*, *VE-cadherin*, and C*D31* as reference genes. The following primer sequences were used:

*Notch4*: 5^′^-ctctgcagccctggctatac-3^′^, 5^′^-ggcatcgagcagtgtgtg-3^′^;

*Tie2*: 5^′^-atgcccttctccaccctctcc-3^′^, 5^′^-ccactacctactagtgaagaa-3^′^;

*Cd31*: 5^′^-ctcctcggcgatcttgctgaa-3^′^, 5^′^-gtcatggccatggtcgagta-3^′^;

*VE-cadherin*: 5^′^-gtaagtgaccaactgctcgtgaat-3^′^, 5^′^-tcctctgcatcctcactatcaca-3^′^

### Immunoprecipitation and western blotting

Immunoprecipitation of Notch4 from lysates of normal mammary gland at pubertal stage followed by Western blotting analysis was performed as previously described [16] using a polyclonal rabbit anti-Notch4 antibody (Upstate).

### Statistical analysis

Vessel density and perfusion analysis was performed as previously described [13]. Data are expressed as mean + standard error of the mean (s.e.m). P-values were calculated using a two-tailed t test except for tumor onset analysis where Fisher’s exact test was applied. Values of p≤0.05 were considered statistically significant.

## Results

### Notch4 expression is upregulated in the vasculature of mouse and human mammary tumors

**Figure 1:**
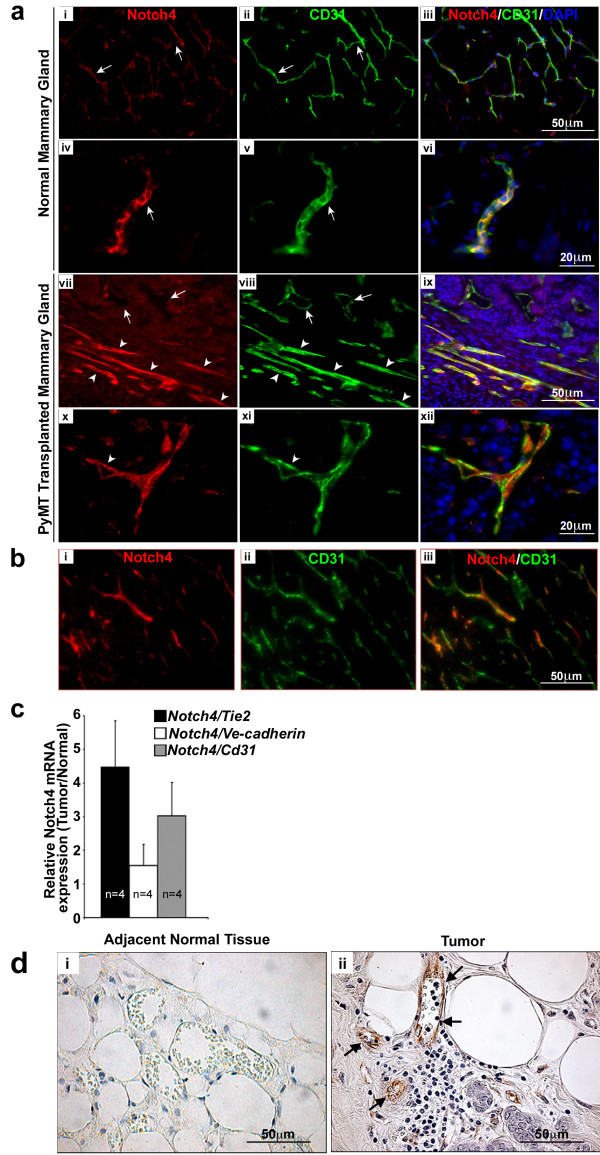
**Notch4 expression is upregulated in the vasculature of mouse and human mammary tumors.** (**a**) Notch4/CD31 immunostaining reveals an increase of Notch4 expression in the vasculature of mouse orthotopic *MMTV-PyMT* mammary tumors (vii-xii) compared to age and stage matched (virgin 8 weeks old) normal mouse mammary glands (i-vi). In panels vii-viii arrows indicate Notch4 low-expressing vessels; arrowheads indicate Notch4 positive vessels. Panels i to iii show low expression levels of Notch4 and CD31 in the vessels of normal mammary gland. Panels iv to vi show a detail of the staining of Notch4 and CD31 in the vessels of normal mammary gland. Panels vii-xii show Notch4 and CD31 staining in tumors without adjacent normal tissues in these images. Panels vii and ix show higher expression of Notch4 in vessels of the tumors than those in the normal gland (as compared to panels i and iii). Panels x (Notch4 staining), xi (CD31 staining) and xii (merged) show higher magnification of tumor vessels with Notch4 expression. (**b**) Notch4 staining in the tumor is mainly restricted to the vasculature. Orthotropic *MMTV-PyMT* mammary tumors at 3 weeks post transplant were immunostained for (i) Notch4, and (ii) CD31. Panel iii shows the merge of Notch4 and CD31. (**c**) *Notch4* mRNA expression is increased in orthotopic tumors derived from transplanted *MMTV-PyMT* cells compared to normal mammary tissue as measured by quantitative RT-PCR. Data is represented as fold change of *Notch4* expression in tumor over normal mammary glands, normalized to the expression of *Tie2*, *VE-cadherin* and *CD31*. Samples were analyzed in duplicate and error bars represent standard error of mean (s.e.m). (**d**) Notch4 immunostaining (brown stain) is increased in the vasculature of human infiltrating ductal carcinoma tissue (ii) compared to adjacent normal tissue (i). Arrows indicate Notch4 staining in the endothelium.

To test the hypothesis that Notch4 is involved in pathological angiogenesis, we used mammary tumors as a model. We first assessed whether an orthotopic tumor model derived from the *MMTV*-*PyMT (polyomamiddleTantigen* driven by the mouse mammary tumor virus LTR*)* transgenic mouse line is a valid experimental tool to study the functional relevance of Notch4 in breast cancer development and angiogenesis. *PyMT* is a potent oncogene that activates oncogenic signaling pathways frequently upregulated in human breast cancer [17]. In the *MMTV-PyMT* mouse, *PyMT* is under the control of the MMTV LTR, targeting *PyMT* expression to the mammary epithelium. The expression of this transgene leads to tumor development that closely mimics several morphological stages of human breast tumor initiation and progression [18]. We started by comparing Notch4 expression between *MMTV-PyMT* tumors generated by orthotopic transplantation and normal mammary glands. To this end, we injected primary tumor cells (at the carcinoma stage) derived from *MMTV-PyMT* mice into the inguinal mammary fat pads of wild type syngeneic female mice. Three weeks after transplantation, we performed co-immunostaining for Notch4 and CD31, to identify endothelial cells. Mammary glands from non-injected, wildtype mice were stained in parallel as controls (Figure 1a, i-vi). The anatomical location of the vasculature relative to the normal mammary structures has been reported in mice. The two structures are closely associated [19,20]. We found that Notch4 expression in the tumor was mostly restricted to the endothelium (Figure 1a, vii-xii and Figure 1b i-iii), an expression pattern that resembles that in the normal tissues (Figure 1a, i-vi). More importantly, we discovered that Notch4 immuno-reactivity was increased in vessels of the tumor (Figure 1a, vii-xii) relative to that in normal mouse mammary glands (Figure 1a, i-vi). A few cells that did not mark for CD31, but were positive for Notch4 (likely myeloid cells, [21]), were also detected both in the normal mammary gland (Figure 1a, i-vi) and infiltrating the solid tumor epithelium (Figure 1a, x-xii).

To corroborate our immunostaining data, *Notch4* mRNA expression was measured by quantitative RT-PCR in four independent sets of normal mouse mammary glands and tumors derived from *MMTV-PyMT* cells transplanted into contra-lateral mammary glands. Since vessel density is known to be increased in the mammary glands of tumor-bearing mice [22], *Notch4* mRNA levels were expressed relative to three endothelium-specific genes: *Tie2*, *VE-cad*, and *CD31*. We found that the levels of *Notch4* mRNA were increased in the mouse mammary tumor tissue relative to normal glands (Figure 1c).

Next, we examined human biopsy samples (n=6) from infiltrating ductal carcinoma, which were immunostained with an antibody against Notch4. We found increased Notch4 expression specifically in the tumor vasculature (Figure 1d, ii) relative to blood vessels in the adjacent normal tissue (Figure 1d, i). This result indicates that, consistent with the finding in mice, up-regulation of vascular Notch4 also occurs in human breast tumorigenesis and suggests that host Notch4 may play a role in breast tumor angiogenesis.

Taken together, our results show that Notch4 expression is upregulated in the tumor vasculature in both murine and human mammary tumors, suggesting a possible role for this receptor in breast cancer formation and angiogenesis.

### Tumor onset is delayed in the absence of host Notch4, along with poor vessel perfusion

**Figure 2:**
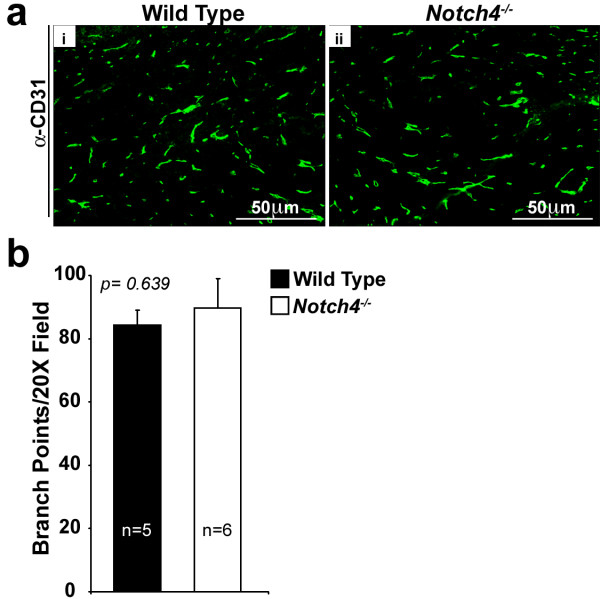
**Vessel density is comparable in *Notch4*-deficient and wild type mouse mammary glands.** (**a**) CD31 immunostaining (green) pattern is similar in wild type (i) and *Notch4−/−* (ii) mouse mammary glands. (**b**) Vessel density (represented as branch points/20X field in intact inguinal mammary fat pads) is similar in wild type and *Notch4−/−* mice. Bars represent average values and error bars represent s.e.m.

To study the function of Notch4 during angiogenesis in mammary tumors, we took advantage of the orthotopic tumor model described above. This model lets us test host Notch4 effects independently of tumor Notch4 effects, because tumor cells from a *Notch4*^*+/+*^ mouse can be transplanted into a syngeneic *Notch4*^*−/−*^ mouse. We verified that the Notch4 protein was absent in *Notch4*^*−/−*^ mice by performing immunoprecipitation and western blot analysis on mammary tissue lysates from wild type, *Notch4*^*+/−*^, and *Notch4*^*−/−*^ mice. As expected, we observed deletion of Notch4 at the protein level in mammary glands from *Notch4*^*−/−*^ mice (Additional file 1: Figure S1). A previous study showed that mammary glands isolated from virgin, pregnant, and lactating *Notch4*^*−/−*^ female mice display no detectable morphological or histological defects, but the mammary gland vasculature in these mice was not characterized [3]. To examine a possible role for Notch4 in mammary vascular morphogenesis, we compared the vessel density in the mammary gland between wild type and *Notch4*^*−/−*^ mice at 3 weeks of age, same age at which mice were transplanted with tumor cells. Vessel density was analyzed by immunostaining for CD31 and counting the number of branch points (the point at which one vessel segment splits into two or more vessel segments). We found that vessel density in *Notch4*^*−/−*^ mammary glands was unaffected by the mutation (Figure 2a, i-ii and 2b). Our results, together with those from previously published studies, suggest that the mammary vasculature develops normally in the absence of Notch4.

**Figure 3:**
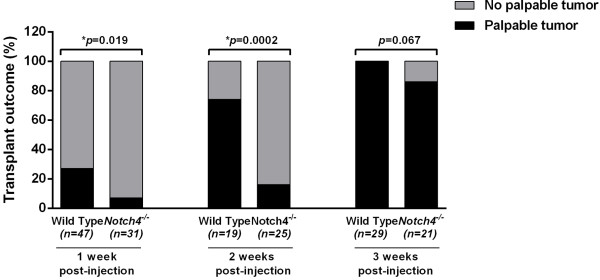
**Host Notch4 deficiency delays onset of tumors derived from orthotopic transplantation of *MMTV-PyMT *cells.** Incidence of palpable tumors in wild type and *Notch4−/−* mice at 1, 2 and 3 weeks post orthotopic transplantation of *MMTV-PyMT* tumor cells. Tumor onset is significantly delayed in *Notch4−/−* mice at the early time points.

We next compared the onset of palpable tumors in wild type and *Notch4*^*−/−*^ mammary glands upon orthotopic injection of syngeneic primary *MMTV-PyMT* tumor cells wild type for *Notch4*. We designate the appearance of palpable tumors after injection of single cells dissociated from carcinoma tissues as tumor onset (hereafter referred to as such). Tumor onset in orthotopic transplants cannot completely mimic natural tumor progression, but it does allow us to determine the contributions from the tumor *vs*. host microenvironment and to explore the tumor-host interactions that are critical in tumor growth and progression. We scored the transplanted mice for incidence of tumor onset at different times after transplantation. We found that tumor onset was significantly delayed in *Notch4*^*−/−*^ hosts compared to wild type controls. One week after transplantation, tumor incidence was 27% in wild type hosts, but only 7% in *Notch4*^*−/−*^ hosts, and two weeks after transplantation, tumor incidence was still significantly reduced in the *Notch4*^*−/−*^ hosts (Figure 3). Three weeks after transplantation, 100% of wild type hosts bore tumor whereas 86% of *Notch4*^*−/−*^ hosts presented tumors, although this difference was not statistically significant (Figure 3). From the very few tumors that were detected at one week after transplantation, we found that the average tumor mass was 0.018±0.004 (s.d.) grams in five tumors developed in wild type hosts. However, the tumors developed in *Notch4*^*−/−*^ hosts were too small to be measured. Therefore, our results reveal that host Notch4 plays a necessary role in tumor onset.

**Figure 4:**
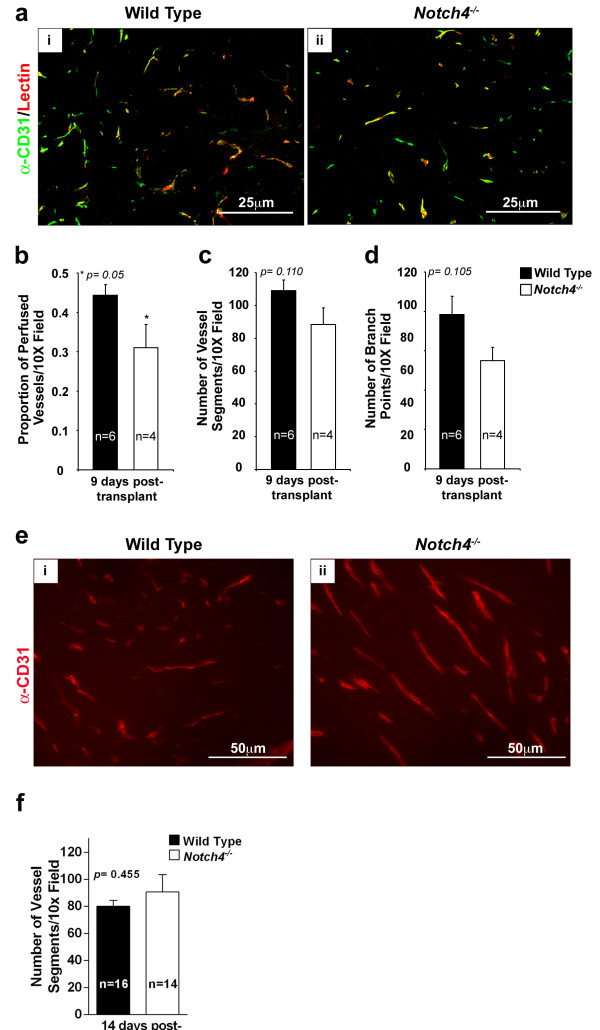
**Vascular perfusion is compromised in tumors developed in *Notch4***^***−/−***^**hosts whereas vessel density is unaffected.** (**a**) CD31 immunostaining (green) and lectin-perfused (red) blood vessel patterns in orthotopic *MMTV-PyMT* tumors are similar in wild type (i) and *Notch4*^−/−^ (ii) hosts. (**b**) Vessel perfusion in *MMTV-PyMT* tumors is decreased in *Notch4*^*−/−*^ hosts compared to wild type hosts 9 days after orthotopic transplantation. (**c** and **d**) Vessel density, either measured as number of vessel segments (**c**) or as number of branch points (**d**) in *MMTV-PyMT* tumors is similar in wild type and *Notch4*^*−/−*^ hosts 9 days after orthotopic transplantation. (**e**) CD31 staining in tumors developed in wild type (i) and *Notch4*^*−/−*^ (ii) hosts 2 weeks after transplantation. (**f**) Vessel density in *MMTV-PyMT* tumors is similar in wild type and *Notch4*^*−/−*^ hosts 2 weeks after orthotopic transplantation. Bars represent average values and error bars represent s.e.m.

Given that Notch4 is predominantly expressed in the normal endothelium and upregulated in the tumor vasculature, we examined blood vessels of tumors transplanted into wild type and *Notch4*^*−/−*^ mice. We performed this analysis by one week after transplantation, when there is a large difference in tumor onset between the two host genotypes (Figure 3). Wild type and *Notch4*^*−/−*^ mice received intravenous injections of Cy3-lectin to highlight vessel perfusion, and tumor sections were immunostained with antibodies against CD31 to highlight overall tumor vasculature (Figure 4a). We found that the number of perfused vessel segments in tumors of *Notch4*^*−/−*^ hosts decreased significantly compared to those of wild type hosts (Figure 4b). Surprisingly, we did not detect any statistically significant differences in the total number of vessel segments and branch points between tumors developed in wild type and *Notch4*^*−/−*^ hosts (Figure 4c and d). To confirm that vessel density is similar between tumors developed in wild type and *Notch4*^*−/−*^ hosts, we quantified vessel segments at 14 days after transplantation when tumor incidence is still significantly reduced (Figure 3). At this time point, we also did not detect any significant differences in vessel density between the two groups (Figure 4e and f). These findings suggest that host Notch4 is required for adequate initial tumor vessel perfusion, but dispensable for vessel sprouting.

**Figure 5:**
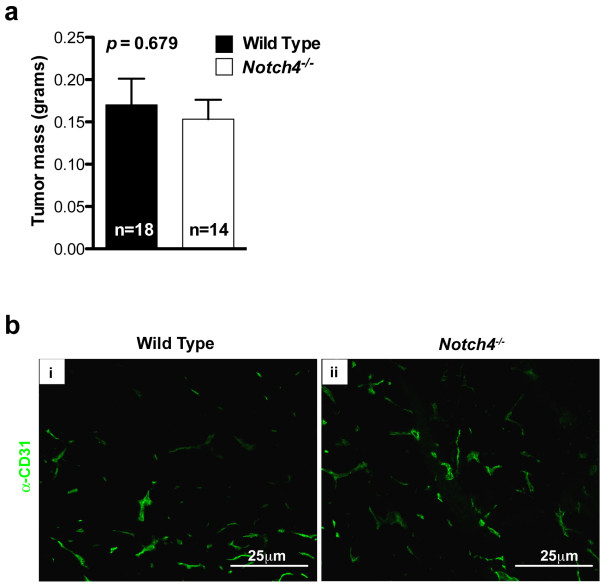
**Tumor mass and vascular architecture are similar in tumors developed in wild type and *Notch4***^***−/−***^**hosts at later stages.** (**a**) Tumor mass of *MMTV-PyMT* tumors at 3 weeks after orthotopic transplantation. Bars represent average values and error bars represent s.e.m. (**b**) CD31 staining highlighting tumor vascular architecture 3 weeks after orthotopic transplantation.

Next we examined whether host Notch4 was required for tumor progression after tumor onset. Tumor-bearing wild type and *Notch4*^*−/−*^ mice were dissected three weeks after transplantation, when tumor incidence is comparable between the two host genotypes. At this stage, tumor mass did not differ significantly between wild type and *Notch4*^*−/−*^ hosts (Figure 5a) and the vascular architecture was also similar (Figure 5b). These results suggest tumors can eventually grow in *Notch4*^*−/−*^ hosts.

## Discussion

In this study we demonstrate that Notch4 expression is upregulated in the vasculature of mammary tumors. Furthermore, our results suggest that host Notch4 plays a role in the early emergence of *MMTV-PyMT* breast tumors following transplantation. We also found that host Notch4 is required for initial tumor vascular perfusion, but not vessel sprouting. To our knowledge, this is the first report demonstrating the functional relevance of host Notch4 upregulation in tumorigenesis and tumor vessel perfusion. Our findings shed light on the role of Notch4, independent of other Notch receptors, in tumor-host interactions.

Our results show increased levels of Notch4 in the blood vessels of mouse and human breast tumor tissues. Expression of Notch receptors and ligands has been described in a wide variety of different tumor types, such as colorectal, prostate, liver, pancreatic and breast cancer [23,24] and a role for Notch4 in regulating breast cancer stem cell activity has been proposed [25]. Mittal *et al.* reported that the levels of Notch receptors (1, 2, and 4) and ligands (Jagged 1, 2, Dll1, and 4) are increased in human breast cancer compared to normal breast tissue [26]. Recently, a study by Speiser and collaborators showed increased Notch1 and Notch4 levels in tumor epithelial cells and vascular endothelial cells in triple-negative breast cancer samples [27], therefore highlighting the relevance of Notch4 expression in the vasculature. However, in our studies, we did not observe Notch4 staining in either human (Figure 1c) or murine tumor cells (Figure 1a, ix, xii and 1b, iii), but rather observed Notch4 upregulation in the tumor vasculature. We used a well-characterized Notch4-specific antibody [28] and verified its specificity using Notch4 knockout tissues (Additional file 1: Figure S1). Our study demonstrates Notch4 upregulation in the vasculature of both mouse models of mammary adenocarcinoma and human breast cancer. It is possible that different types and grades of breast carcinoma present different Notch4 expression levels and distribution. However, in our study, our mouse model provides evidence that host, likely vascular, Notch4 plays a role in breast cancer development. Consistent with our finding that Notch4 is expressed in the tumor vasculature, the Notch ligand Dll4 is detected in the vessels of infiltrating human breast adenocarcinoma samples [29], making it a possible ligand for Notch4 in tumor vasculature.

Orthotopic transplantation of mammary tumor cells is a well-established model for *in vivo* studies of breast tumorigenesis, and we chose this approach because it allowed us to study host Notch4-mediated effects on tumorigenesis independent of Notch4 activity in the tumor cell compartment. We demonstrate a contribution of the tumor microenvironment, namely the host Notch4, to tumorigenesis. We also pinpoint that the tumorigenic defect lies at the tumor onset following transplantation*.* It is well documented that the growth of solid tumors depends on the development of new vasculature [30]. Inhibition of members of the Notch signaling pathway leads to an increase in “non-productive” angiogenesis, characterized by a reduction in vessel perfusion despite an increase in vessel sprouting [9-11,31]. We thus examined vascular perfusion in tumors grown in *Notch4*^*−/−*^ hosts. Since both the mammary gland and its vasculature appear normal in *Notch4*^*−/−*^ mice, we reasoned that any vascular defects in tumors from *Notch4*^*−/−*^ hosts must be due to abnormalities that occur during tumorigenesis, and not as a result of preexisting vascular defects. We observed that vessel perfusion was reduced in tumors grown in *Notch4*^*−/−*^*vs.* wild type hosts. This observation is consistent with many reports of Notch pathway inhibition leading to reduced perfusion [9-11,31].

Although poor tumor vessel perfusion correlated significantly with delayed tumor onset at early time points after transplantation, our results alone do not provide causal proof that reduced vessel perfusion leads to delayed tumor onset in *Notch4*^−/−^ mice. Given the complex dynamics in tumor-host interaction and tumor microenvironment, we cannot rule out the possibility that vascular Notch4 (and other Notch pathway proteins) may regulate tumor onset by mechanisms that are independent of vessel perfusion. It is also possible that differences in transplantation-associated immune responses contribute to the delayed tumor onset in *Notch4*^*−/−*^ hosts. Both tumor-associated fibroblasts and tumor-infiltrating leukocytes have been shown to play an important role in tumor onset and growth [32]. Notch4 expression has been detected in immune cells of myeloid lineage [21]. Although we detected Notch4 overexpression predominantly in the tumor vasculature, we cannot rule out the possibility that host myeloid cells may contribute to the difference in tumor onset between the two host genotypes.

Host Notch4 deficiency delays tumor onset and decreases initial perfusion, however, the growth of established tumors can ultimately progress in the absence of host Notch4. This result suggests that host Notch4 plays a unique role in the initiation of tumor onset after transplantation.

Surprisingly, our results indicate that Notch4 is dispensable for vessel sprouting in the tumor. Sprouting angiogenesis is a hallmark of tumor neovascularization, and Dll4/Notch1 signaling functions to inhibit vessel sprouting [8]. Moreover, given that Notch1 appears to be the primary Notch receptor responsible for developmental angiogenesis [3], together with the results obtained using specific anti-Notch1 antibodies in tumors [11], Notch1 seems to be the predominant mediator of Notch signaling in tumor angiogenesis. It is therefore likely that the increased vascular network observed when inhibiting pan-Notch signaling or Notch ligands is mainly due to the inhibition of Notch1. Alternatively, it is possible that a subtle defect in vessel sprouting exists in the *Notch4*^−/−^ tumor vasculature, but current methodologies are not sensitive enough to detect such subtle phenotype.

## Conclusion

Our finding that the lack of *Notch4* does not increase vascular density, but does affect tumorigenesis and vessel perfusion suggests a unique mechanism distinct from that mediated by Notch1. Our work uncovers a novel role of Notch4 in the early establishment of vessel perfusion; whether this is a mechanism unique to tumor vessel angiogenesis or whether it is present also during development or in other postnatal angiogenic settings remains to be determined.

### Availability of supporting data

The data set supporting the results of this article is included within the article (and its Additional file 1: Figure S1).

## Competing interests

The authors declare to have no competing interests.

## Authors’ contributions

RAW, MJC, XW conceived and designed the experiments; MJC, XW, SKB and EC performed the experiments; MJC, XW, HC, RS, RAW, OM analyzed the data; TG contributed reagents/materials/analysis tools for this manuscript; HC, RAW, CAC, MJC, RS, OM wrote the manuscript. MJC and XW have contributed equally to this manuscript. All authors read and approved the final manuscript.

## Supplementary Material

Additional file 1: Figure S1 Mammary glads from Notch4-/-mice exhibit no detectable Notch4 protein expression. Notch4 immunoprecipitation-immunoblot analysis on mammary tissue lysates from wild type (lane 1), Notch+/- (lane 3), and Notch4-/- (lane 5) mice shows a lack of Notch4 protein in Notch4-/- mice. Lanes 2, 4 and 6 are the same samples immunoprecipiated with a control rabbit lαG. Click here for file
