# Supplementary material for: Notch4 is required for tumor onset and perfusion
Source: Vasc Cell. 2013 Apr 20;5:7. doi: 10.1186/2045-824X-5-7 (PMC3644271; doi:10.1186/2045-824X-5-7)
Supplement: Additional file 1: Figure S1 — Mammary glads from Notch4-/-mice exhibit no detectable Notch4 protein expression. Notch4 immunoprecipitation-immunoblot analysis on mammary tissue lysates from wild type (lane 1), Notch+/- (lane 3), and Notch4-/- (lane 5) mice shows a lack of Notch4 protein in Notch4-/- mice. Lanes 2, 4 and 6 are the same samples immunoprecipiated with a control rabbit lαG. [file 2045-824X-5-7-S1.pdf]

Supplementary Figure 1

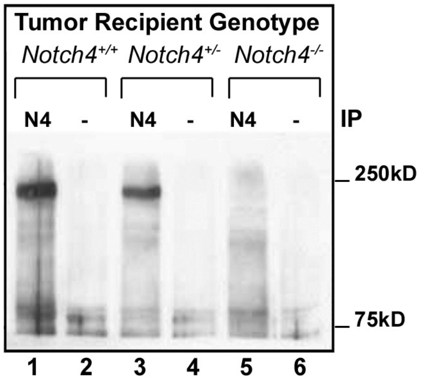

Supplementary Figure 1: Mammary glands from Notch4-/- mice exhibit no detectable Notch4 protein expression. Notch4 immunoprecipitation-immunoblot analysis on mammary tissue lysates from wild-type (lane 1), Notch4+/- (lane 3), and Notch4-/- (lane 5) mice shows a lack of Notch4 protein in Notch4-/- mice. Lanes 2, 4 and 6 are the same samples immunoprecipiated with a control rabbit IgG.
